# A zebrafish reporter line reveals immune and neuronal expression of endogenous retrovirus

**DOI:** 10.1242/dmm.048921

**Published:** 2022-04-13

**Authors:** Holly A. Rutherford, Amy Clarke, Emily V. Chambers, Jessica J. Petts, Euan G. Carson, Hannah M. Isles, Alejandra Duque-Jaramillo, Stephen A. Renshaw, Jean-Pierre Levraud, Noémie Hamilton

**Affiliations:** 1 The Bateson Centre, Department of Infection, Immunity and Cardiovascular Disease, University of Sheffield, Sheffield S10 2TN, UK; 2 The Bioinformatics Core, Faculty of Medicine and Dentistry, University of Sheffield, Sheffield S10 2TN, UK; 3 Institute of Microbiology (IMUL), Lausanne University Hospital and University of Lausanne, Rue du Bugnon 48, 1011 Lausanne, Switzerland; 4 Macrophages et Développement de l'Immunité, Institut Pasteur, CNRS UMR3738, 25 Rue du Docteur Roux, 75015 Paris, France; 5 The Institute of Neuroscience, University of Sheffield, Sheffield S10 2TN, UK

**Keywords:** Retroelement, Zebrafish, Endogenous retrovirus, Reporter line, *zferv*, LTR

## Abstract

Endogenous retroviruses (ERVs) are fossils left in our genome from retrovirus infections of the past. Their sequences are part of every vertebrate genome and their random integrations are thought to have contributed to evolution. Although ERVs are mainly silenced by the host genome, they have been found to be activated in multiple disease states, such as auto-inflammatory disorders and neurological diseases. However, the numerous copies in mammalian genomes and the lack of tools to study them make defining their role in health and diseases challenging. In this study, we identified eight copies of the zebrafish endogenous retrovirus *zferv*. We created and characterised the first *in vivo* ERV reporter line in any species. Using a combination of live imaging, flow cytometry and single-cell RNA sequencing, we mapped *zferv* expression to early T cells and neurons. Thus, this new tool identified tissues expressing ERV in zebrafish, highlighting a potential role of ERV during brain development and strengthening the hypothesis that ERV play a role in immunity and neurological diseases. This transgenic line is therefore a suitable tool to study the function of ERV in health and diseases.

## Abstract (precis)

**Summary:** Characterisation of the zebrafish endogenous retrovirus *zferv* reveals expression in early T cells and neurons, highlighting a potential role of endogenous retroviruses in brain development and immune disorders.

## INTRODUCTION

Over 40% of the human genome consists of endogenous transposable elements capable of recombination and disruption of genes and modification of their expression (Lander et al., 2001). Endogenous retroviruses (ERVs) are transposable elements originating from old integrations of retroviruses so successful that they have become part of most vertebrate genomes studied. ERVs replicate autonomously using a ‘copy-and-paste’ mechanism and, although they constitute a smaller percentage of all retroelements, they still represent 8% of the human genome (Bourque et al., 2018; Lander et al., 2001). The majority of known ERVs have lost their ability to replicate, but those most recently acquired still have intact genomes with the ability to produce viral RNA and particles. However, many competent ERVs are under strict transcriptional suppression by epigenetic mechanisms (Maksakova et al., 2008; Rowe et al., 2010; Turelli et al., 2014), protecting the host organisms against potential retroviral insertions and viral activities.

Although immobilised by mutations or transcriptionally repressed, ERVs have a complex relationship with the human genome, which they can regulate by providing cis-regulatory elements to surrounding genes and by lifting their transcriptionally repressed state. Through these mechanisms, it is believed that transposable elements have fuelled some of the necessary genetic changes for evolution (Feschotte, 2008; Kunarso et al., 2010). Syncytin-1, an ERV envelope gene essential for the vascularisation of the placenta, is at the origin of evolutionary diversification of the placenta (Chuong, 2018; Mi et al., 2000). During human embryogenesis, expression of specific ERV families have been associated with cell identity and cell potency in early stem cells (Göke et al., 2015). Additionally, ERV expression has been reported in healthy human tissues, such as ovary and testis for ERV-9 (Pi et al., 2004), pancreas (Shiroma et al., 2001), breast (Tavakolian et al., 2019), stomach and small intestine (Okahara et al., 2004). Mainly based on transcriptional studies, the expression of different families of ERV is likely to be extended to more tissues; however, their role in tissue development and function remains largely unknown.

ERVs have been linked directly and indirectly to the evolution and functioning of the immune system. Enhancer regions of interferon-stimulated genes key to the interferon pathway, such as *IRF1* and *STAT1*, were found introduced and amplified by ERV elements, with the human inflammasome failing to form upon the deletion of a subset of ERVs (Chuong et al., 2016). Adaptive immunity also benefits from the presence of ERVs. Indeed, ERV peptide recognition is used in T-cell selection to optimise antigen recognition and T cells have a higher sensitivity to exogenous virus infection when presented with ERV peptides during their initial thymic selection (Mandl et al., 2013; Young et al., 2012). The human ERV (HERV) envelope gene contains immunosuppressive domains that reduce the Th1 response during pregnancy and therefore promote foetal development (Knerr et al., 2004; Lokossou et al., 2020). The role of ERVs in our immune system, particularly in the training of our adaptive immunity, can be a double-edged sword as ERVs are linked to a range of different disease states, including autoimmunity.

Aberrant expression of ERVs contributes to multiple pathologies. ERVs are found in abundance in multiple forms of cancers and are considered tumour-promoting factors (extensively reviewed by Bermejo et al., 2020). The pathology of autoinflammatory diseases has also been strongly associated with ERVs. Systemic lupus erythematosus (SLE) is an autoimmune disorder with increased levels of autoantigen for an ERV (Baudino et al., 2014). Recently, one murine SLE susceptibility locus was identified as a key suppressor of ERV expression, consolidating the role of ERVs in the pathogenesis of SLE (Treger et al., 2019). A similar disorder is Aicardi–Goutières Syndrome (AGS), a type 1 interferonopathy that resembles congenital cytomegalovirus infection and is caused by mutations in several genes encoding enzymes responsible for nucleic acid metabolism (Crow et al., 2015). Mutations in some of these genes, such as *TREX1*, *MDA5* (*IFIH1*) and *ADAR1* (*ADAR*), trigger an antiviral immune response due to self-reactivity of retroelements (Ahmad et al., 2018; Benitez-Guijarro et al., 2018; Chung et al., 2018; Li et al., 2017; Thomas et al., 2017). Interestingly, anti-reverse transcriptase therapy in AGS patients can decrease the interferon response, highlighting the role of aberrant presence of ERVs in triggering an immune response (Rice et al., 2018). Increased expression of ERVs has been found in brains of patient suffering from neurodegenerative diseases, such as motor neuron disease (Li et al., 2015) and multiple sclerosis (MS) (Johnston et al., 2001; Mameli et al., 2007). Overexpression of a human ERV in neurons was shown to be neurotoxic, suggesting a potential role of ERVs in triggering neuronal toxicity (Li et al., 2015). A direct link to the pathology of these disorders has yet to be made, but, nonetheless, ERVs appear to be strong causal factors for autoimmune and neurological disorders.

Although ERV enrichment has been detected in neurological pathologies, little is known about the function of ERV in healthy tissues. The exact role of ERV in our immune system and brain pathologies has yet to be understood and there is no model system specifically looking at ERV function *in vivo*. Zebrafish is already established as a model to study the immune system, with significant homology with mammals in innate and adaptive immunity (Renshaw and Trede, 2012; Trede et al., 2004). The genetic tractability and transparency of the zebrafish embryos have allowed the creation of transgenic reporter lines, some of which have elucidated the role of immune cell behaviour *in vivo* (Renshaw et al., 2006). The tractability of the zebrafish has already been exploited to visualise the expression of the human ERV-9 in oocytes, similarly to human expression (Pi et al., 2004).

In this study, we used the zebrafish as a tractable *in vivo* model to characterise the zebrafish endogenous retrovirus *zferv*. We identified multiple *zferv* family members, including two complete genomes, we named *zferv1a* and *zferv1b*. Using the transparency of the zebrafish larvae, we developed a reporter line for *zferv1a* and imaged for the first time ERV activation in healthy tissues *in vivo*. We showed that *zferv1a* is expressed in the thymus and in the brain. Colocalisation analysis and single-cell RNA sequencing revealed expression of *zferv1a* specifically in T cells, suggesting a potential role for ERV in lymphocyte function or development. Brain expression of *zferv1a* appears to include neuronal expression. This transgenic line can be used as a tool to investigate further the role of ERVs in immunity and in neurological disorders.

## RESULTS

### The zebrafish genome contains multiple endogenous retrovirus integrations

**Fig. 1.**
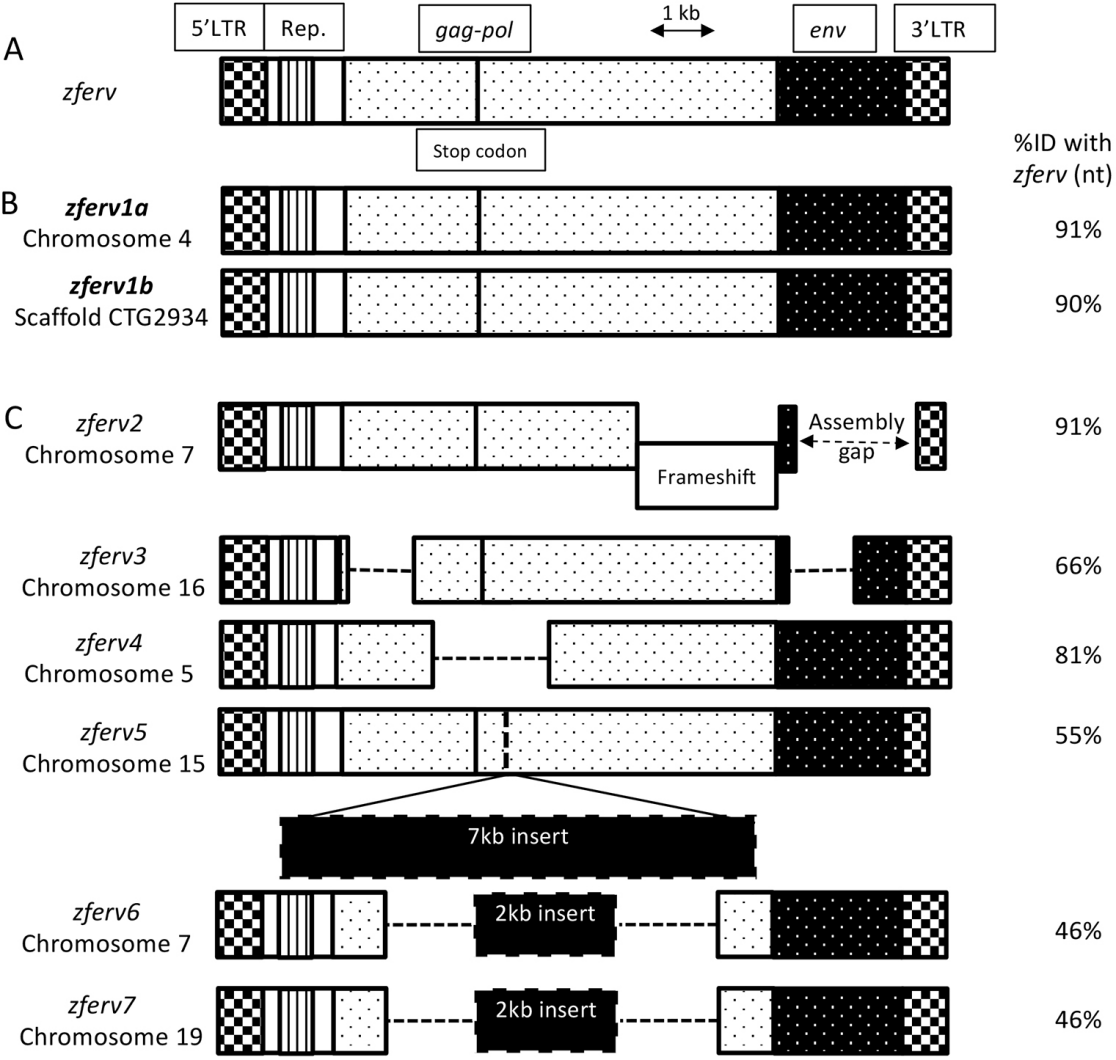
**Multiple copies of *zferv* are present in the zebrafish genome.** (A) Diagram of the original *zferv* genes found by Shen and Steiner (2004) used as a reference for nucleotide identity (ID% nt). (B) Diagram representing the two closest related *zferv* genes found in most recent zebrafish genome GRCz11, named *zferv1a* and *zferv1b*. (C) Diagram of six pseudo *zferv* genes with degenerated genome (dashed line represents insertions). 3′LTR, 3′ long terminal repeat; 5′LTR, 5′ long terminal repeat; *env*, envelope gene; *gag-pol*, genes encoding polyprotein and reverse transcriptase; Rep., repetitive element.

The presence of an ERV in zebrafish, named *zferv*, was reported by Shen and Steiner while screening a thymic cDNA library (Shen and Steiner, 2004) (Fig. 1A). We searched for related sequences in the most recent reference zebrafish genome (GRCz11, Tü strain) using BLASTN searches, with the original *zferv* sequence as a query. Limiting ourselves to sequences flanked by long terminal repeats (LTRs) on both sides, we retrieved eight hits scattered in the zebrafish genome, but not the exact *zferv* sequence, possibly because of strain difference (Fig. 1, Table S1). We identified two sequences encoding apparently fully functional ERVs, with 91% and 90% identity to *zferv*, which we named *zferv1a* and *zferv1b*, respectively (Fig. 1B). These two ERVs encode almost identical proteins (95-97% identity at the amino-acid level) and have highly homologous LTR promoter regions (95% identity at the nucleotide level). An additional six pseudo *zferv* genes (here called *zferv2* to *zferv7*) were identified (Fig. 1C), *zferv2* containing a frameshift, *zferv3* and *zferv4* with large deletions, and *zferv5*, *zferv6* and *zferv7* containing a large insertion, all resulting in a degenerated ERV genome.

### *zferv1a* is actively expressed in the brain and in the thymus

**Fig. 2.**
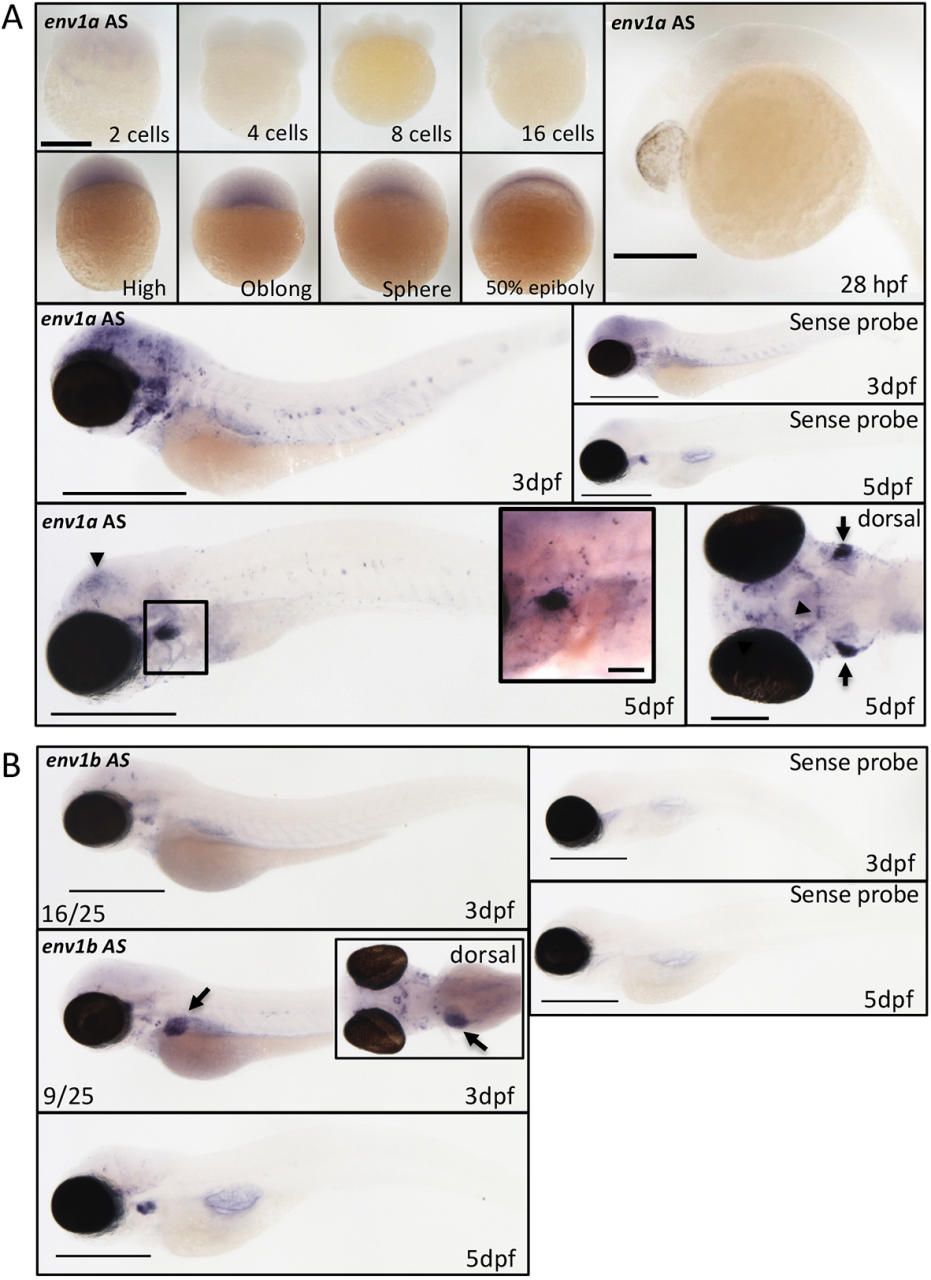
**Reporter line for *zferv1a* recapitulates endogenous expression.** (A) Expression of the envelope gene (*env*) of *zferv1a* (called *env1a* here) using antisense (AS) and sense *in situ* hybridisation RNA probes from the two-cell stage until 5 dpf (black arrowheads point to brain expression). Scale bars: 500 µm unless specified otherwise. Bottom left panel shows dorsal view of *env1aAS* expression in the brain (black arrowheads) and thymus (black arrows). Scale bar: 200 µm. Inset shows a magnified image of the thymus area (boxed), highlighting strong expression around the thymus with single positive cells visible in the vicinity of the thymus around the ear and alongside the branchial arches. Scale bar: 70 µm. (B) Expression of the envelope gene (*env*) of *zferv1b* (called *env1b AS* here) by *in situ* hybridisation at 3 dpf and 5 dpf. Liver expression is highlighted by black arrows. Inset shows dorsal view of the asymmetric liver expression. Scale bars: 500 µm.

*zferv* expression was initially found in the thymus at 5 days post-fertilisation (dpf) using an RNA probe against the envelope (*env*) gene of the original *zferv* (Shen and Steiner, 2004). To verify this observation, the entire *zferv1a* genome was subcloned from a fosmid provided by the Sanger Center (Cambridge, UK) and used to create an *in situ* hybridisation (ISH) RNA probe against the envelope gene of *zferv1a*, here called *env1a*. ISH performed on a time course starting from two-cell-stage embryos until 5 dpf confirmed a strong signal in the thymus at 5 dpf (Fig. 2A), similarly to what was previously reported (Shen and Steiner, 2004). Individually labelled cells were visible around the thymus following the branchial arches and around the ear (Fig. 2A, black box). Additionally, we identified a signal at an earlier time point during gastrulation and a clear signal in the brain, which was particularly strong at 3 dpf (Fig. 2A). Neuromasts were labelled after 24 h exposure to the probe (Fig. S1). There was some signal in the thymus using the sense probe signal, suggesting some bidirectional expression of *zferv1a*, as reported for some human ERVs (Chiappinelli et al., 2015).

To ensure the specificity of this probe to the *zferv1a* gene, we created a probe against the reverse transcriptase gene of *zferv1a* (*pol1a*) and showed similar labelling of the brain and thymus (Fig. S2). We additionally created a probe against another *zferv* isoform, using specific primers (Fig. S3) to the envelope gene of *zferv1b* (called here *env1b*). Although we still identified a signal in the thymus at 5 dpf, we observed a different expression pattern from *env1a* with no brain expression and a strong labelling of the liver in some embryos at 3 dpf (Fig. 2B).

### *zferv1a* reporter line recapitulates endogenous expression

**Fig. 3.**
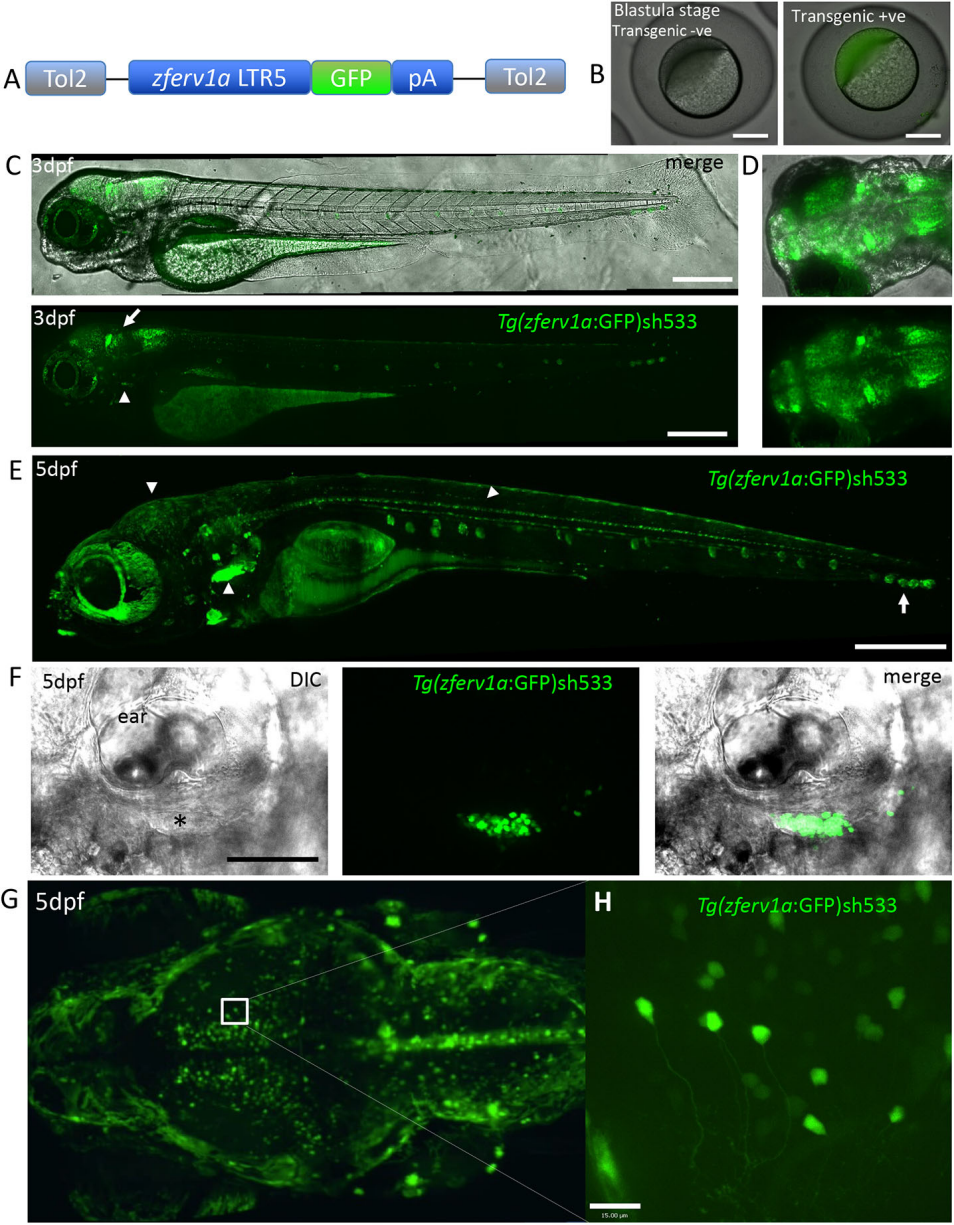
**Reporter line for *zferv1a* recapitulates endogenous expression.** (A) Diagram of the Gateway construct used to create the Tg(*zferv1a:*GFP) reporter line using the pDestCryCFP Tol2 backbone. (B) Representative images of non-transgenic and Tg(*zferv1a:*GFP*:pA*)*sh533* transgenic embryos at the blastula stage. (C) Lateral view of DIC and GFP high-resolution images of a 3 dpf Tg(*zferv1a:*GFP*:pA*)*sh533* embryo, displaying high expression of the transgene in the brain (white arrow) and the start of a signal in the thymus (white arrowheads). Scale bars: 400 µm. (D) Dorsal view of the brain at 3 dpf, showing GFP expression alone or merged with brightfield image. (E) High-resolution image of a 5 dpf Tg(*zferv1a:*GFP*:pA*)*sh533* embryo, displaying high expression of the transgene in the thymus, brain and spinal cord (white arrowheads). Note the signal in neuromasts alongside the trunk (white arrow). Scale bar: 500 µm. (F) Single-slice DIC and confocal GFP fluorescence merged image of the thymus (black asterisk) situated underneath the ear in 5 dpf embryos and the signal from the Tg(*zferv1a:*GFP)*sh533* reporter. Scale bar: 100 µm. (G) Dorsal view of a maximum projection of the brain from a 5 dpf Tg(*zferv1a:*GFP) embryo acquired with a lightsheet microscope. (H) High-resolution single-slice image from the optic tectum of the same Tg(*zferv1a:*GFP) embryo. Scale bar: 15 µm.

To follow the expression of the zebrafish endogenous retrovirus we identified as *zferv1a*, we took a transgenic approach taking advantage of the promoter activity of retroviral LTR. We used the 5′ viral promoter *ltr5* from *zferv1a* to drive GFP expression by Gateway recombination (Fig. 3A). Injected embryos showing expression in their thymus were raised and screened in adulthood for germline transmission; one founder was selected to generate the transgenic line bearing the sh533 allele, which was analysed at the F2 generation. Comparable to observations by ISH, transgenic embryos (obtained from transgenic mothers) displayed GFP signal at the blastula stage and developed a strong GFP signal in the brain by 3 dpf (Fig. 3C,D). GFP signal observed in the eye was caused by the reflective nature of the retina and not by expression of the transgene, as shown using illumination from different laser also highlighting the eye and *in situ* hybridisation labelling with the *env1a* probe in a 5 dpf embryo with pigment partially removed in the eye using a tyrosinase crispant (Fig. S4). A weak signal in the thymus could be observed at 3 dpf (Fig. 3C, white arrowheads) as the thymus started to develop. At 5 dpf, a strong signal was observed in the thymus (Fig. 3E,F), comparable to that observed by ISH (Fig. 2A). Consistent with ISH, our reporter line also labelled the optic tectum region of the brain and the spinal cord at 5 dpf, with axons labelled in individual neurons (Fig. 3G,H). GFP signal was also found in neuromasts (Fig. 3E, white arrow), which we also observed in a small proportion of 5 dpf embryos by ISH with a longer development time (Fig. S1). The transmission rate of 50% to F3 embryos of the brain signal was variable depending on which F2 animal was used, including complete and partial brain labelling, whereas the thymic expression remained constant throughout the generations.

This transgenic line was not the first attempt to create a fluorescent reporter for *zferv1a*. Our initial reporter line was made using both 5′ (*ltr5*) and 3′ (*ltr3*) promoter regions in order to resemble the genome structure of an ERV. This line, called Tg(*ltr5:*GFP*:ltr3*)*sh500*, contained the CryCFP eye marker to help with selection of positive embryos. Although outcrossed F1 animals showed a normal transmission rate (50% of positive embryos in F2, with CFP in lens and GFP in thymus), the F2 transmission rate became unstable. Selected F2-positive embryos grown to adulthood failed to transmit the same signal pattern, with often reduced or complete loss of thymus labelling and random brain expression pattern despite transmission of the CFP lens phenotype at expected Mendelian ratios. We also observed a dampening of the GFP signal in F3 and complete loss in F4 animals. This suggested that the transgene was behaving as a transposable element and was ultimately silenced by the host or lost in the germline. This line was abandoned, and we continued our study with the *sh533* line.

### Zebrafish endogenous retrovirus *zferv1a* expression is restricted to lymphoid cells

**Fig. 4.**
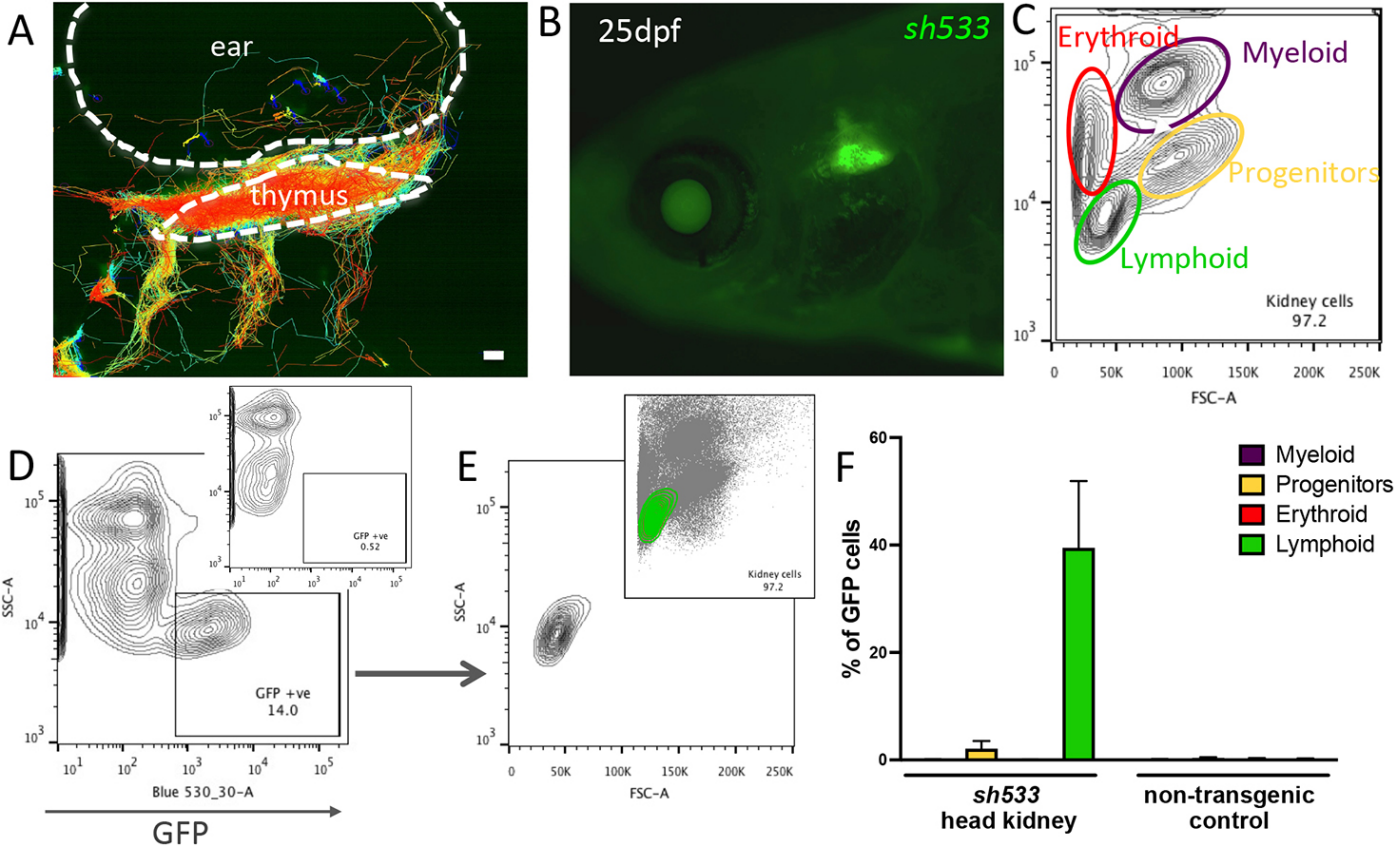
***zferv1a* is expressed in cells from the lymphoid lineage in adult haematopoietic tissues.** (A) Cell tracking reveals dynamic behaviour of entry and exit of the thymus by GFP-positive cells in Tg(*zferv1a*:GFP)*sh533*. Whole-stack analysis of cell movement using TrackMate in Fiji. Scale bar: 10 µm. (B) Expression of Tg(*zferv1a:*GFP)*sh533* in a 25 dpf zebrafish highlighting the triangular shaped thymus. (C) Forward scatter (FSC)/side scatter (SSC) plot of a whole kidney marrow separating different haematopoietic lineages by size and granularity. (D) SSC and GFP plot from a single-cell suspension of a whole kidney marrow from an adult Tg(*zferv1a:*GFP) with gate selecting GFP-positive cells. Inset shows the distribution of cells from a non-transgenic adult whole kidney marrow. (E) FSC/SSC plot of selected GFP-positive cells from D. Inset shows the location of the GFP-positive cells in the haematopoietic lineage FSC/SSC plot. (F) Quantification of GFP-positive cells in myeloid, erythroid, lymphoid lineages and progenitors from dissected *sh533* and non-transgenic control adult head kidneys (*n*=3).

To study further the nature of the cells labelled in the thymus in the *sh533* line, we took advantage of the transparency and ease of access of the thymic tissue to perform live imaging. Cell tracking of time-lapse imaging of the *sh533* line revealed a dynamic behaviour of GFP-labelled cells, approaching and exiting the thymus following the ear and the branchial arches (Fig. 4A, Movie 1), as previously described for thymocytes (Dee et al., 2016; Kissa et al., 2008). This thymic signal persisted beyond embryonic stages and was still visible in juveniles at 25 dpf (Fig. 4B). To analyse the origin of the *zferv1a* cell population, we dissociated three head kidneys from Tg(*zferv1a*:GFP)*sh533* and non-transgenic control adults and used size and granularity scatter by flow cytometry to distinguish between the various haematopoietic lineages (Fig. 4C). According to these criteria and using a back-gating method for quantification (Fig. 4D,E, Fig. S5), we found that GFP-positive cells have a typical lymphocyte profile (Fig. 4F).

**Fig. 5.**
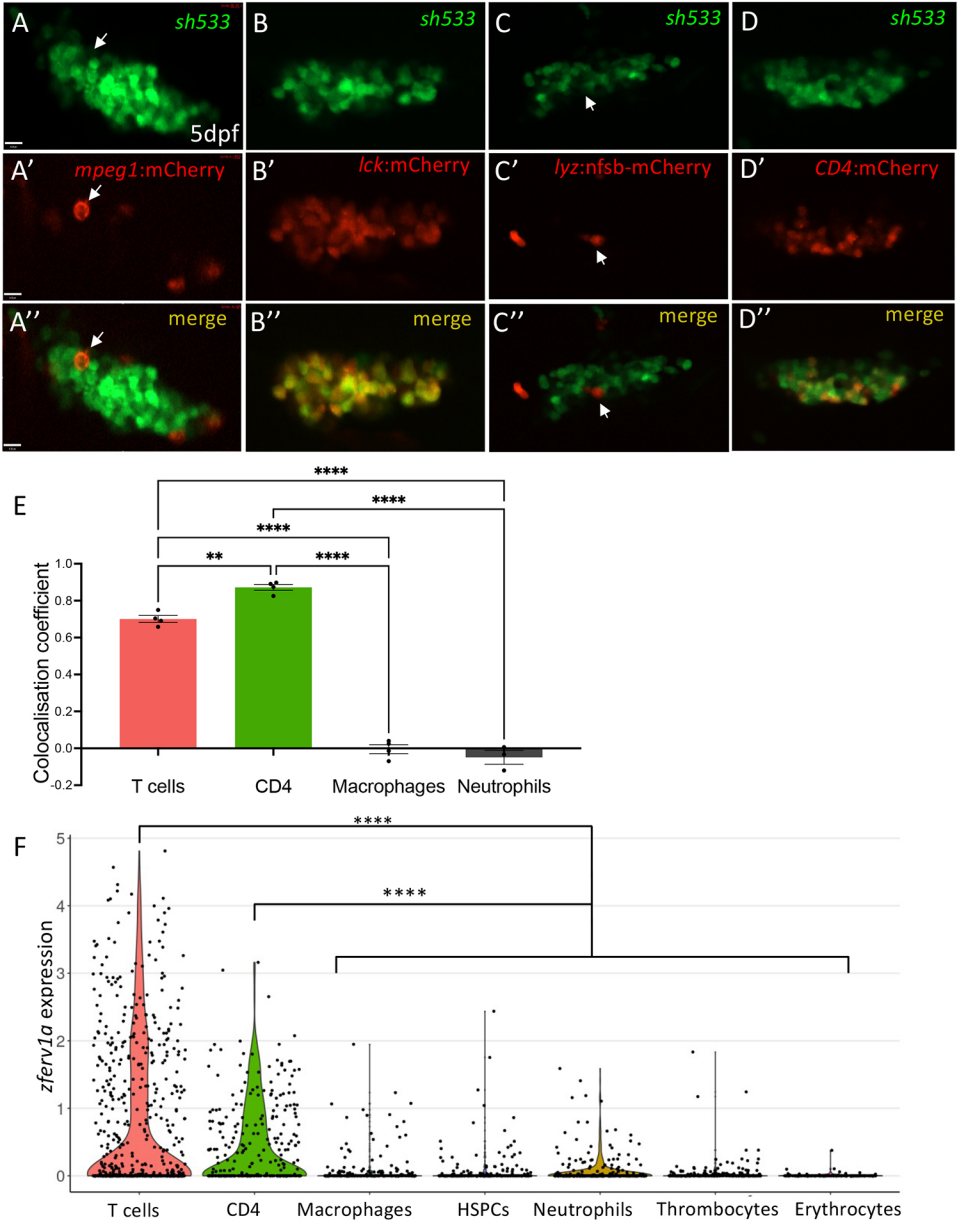
**Immune expression of *zferv1a* is restricted to CD4^+^ cells and T cells.** (A-D″) Single-slice confocal image of the thymus from double-transgenic Tg(*zferv1a:*GFP)*sh533* and Tg(*mpeg1:*mCherryCAAX)*sh378*, which labels macrophage membranes (A-A″, white arrows point to a single macrophage), Tg(*lck:*mCherry), which labels T cells (B-B″), Tg(l*yz:nfsb-*mCherry)*sh260*, which labels neutrophils (C-C″, white arrows point to a single neutrophil), and Tg(*CD4:*mCherryCAAX), which labels CD4^+^ cells (D-D″) with merge image used for colocalisation analysis. (E) Histogram of ImageJ-generated colocalisation coefficients of Tg(*zferv1a:*GFP)*sh533* crossed to Tg(*mpeg1:*mCherryCAAX)*sh378* for macrophages, Tg(*lyz:nfsb-*mCherry)*sh260* for neutrophils, Tg(*CD4:*mCherry) for CD4^+^ cells and Tg(*lck:*mCherry) for T cells (one-way ANOVA, *n*=4). (F) Violin plot of single-cell RNA-sequencing datasets MTAB-5530 and E-MTAB-4617 from zebrafish adult whole kidney marrow and spleen showing expression of *zferv1a* (*ENSDARG00000110878*) in multiple haematopoietic lineages. *P*=1.95×10^−11^ for CD4^+^ versus the other clusters and *P*=1.97×10^−45^ for T cells versus the other clusters (Mann–Whitney with paired Wilcoxon test). HSPCs, haematopoietic stem and progenitor cells.

To confirm the lymphoid identity of *zferv1a*-expressing cells, we performed *in vivo* colocalisation analysis in 5 dpf embryos by crossing the Tg(*zferv1*a:GFP)*sh533* line to different reporter lines labelling a wide spectrum of immune cell populations. We crossed F1 Tg(*zferv1a*:GFP)*sh533* to reporter lines labelling lymphoid cells, such as Tg(*lck*:mCherry), labelling T cells (Langenau et al., 2004) as well as natural killer (NK) and innate lymphoid cells (Hernández et al., 2018), or Tg(*CD4*:mCherry), labelling CD4^+^ T cells and some macrophage/dendritic cells (Dee et al., 2016), and showed significant colocalisation between GFP- and mCherry-expressing cells by colocalisation analysis (Fig. 5B-B″,D-E). By contrast, crossing of Tg(*zferv1a*:GFP)*sh533* to macrophage Tg(*mpeg1*:mCherryCAAX)*sh378* (Fig. 5A-A″) or neutrophil Tg(*lyz:nfsb-*mCherry)*sh260* (Fig. 5C-C″) (Buchan et al., 2019) reporter lines did not reveal significant colocalisation (Fig. 5E). To validate these results, we used single-cell RNA-sequencing datasets available on zebrafish blood cells (ArrayExpress accession numbers: MTAB-5530 and E-MTAB-4617) as a robust assay to analyse gene expression in specific cell types and tissues. Using the extensive gene expression dataset available, we mapped the expression of the entire genome of *zferv1a* (*ENSDARG00000110878*) in erythrocytes, haematopoietic stem and progenitor cells, macrophages, neutrophils, CD4^+^ cells, T cells and thrombocytes. Only sporadic expression was observed in all cell lineages except for the T-cell subsets ‘CD4^+^’ and ‘T cells’, where significant expression of *env* was detected (Fig. 5F). Altogether, these data are consistent with immune expression of *zferv1a* being restricted to T cells.

### Modulation of *zferv1a* expression

To investigate whether *zferv1a* can affect the development of the tissues where it is expressed, we performed an overexpression assay. We injected a vector containing *zferv1a* alongside the same vector containing the eGFP cassette to use as a positive control. Each plasmid was injected in equimolar amount or GFP alone and the expression of *zferv1a* and GFP was measured by qPCR. Overexpression of GFP resulted in a high level of GFP transcript detected by qPCR, whereas no expression of *zferv1a* was detected (Fig. S6). We then checked for induction of *zferv1a* (*ENSDARG00000110878*) in our datasets of larvae stimulated with type I interferon or with chikungunya (CHIKV) virus (Levraud et al., 2019; NCBI BioProject accession number: PRJNA531581). No expression was detected in the dataset of type I interferon-injected larvae (3 dpf). Expression was detected in the dataset of CHIKV-infected fish (4 dpf). However, although CHIKV infection triggers a very strong interferon response, with 1000-fold induction of some interferon-stimulated genes (ISGs), there was only a 1.7-fold increase for *zferv1a*, which was not statistically significant.

## DISCUSSION

We describe here a novel reporter line in zebrafish that allows the expression of an endogenous retrovirus to be followed in real time *in vivo*. Our results validate and refine previously published expression data of *zferv* in the thymus and specifically in the lymphoid lineage. We identified a close relative to *zferv*, here named *zferv1a*, and showed transcriptional activity mostly restricted to T lymphocytes, as previously reported (Frazer et al., 2012; Shen and Steiner, 2004), but also observed in neurons.

### Stability of the engineered-ERV transgene

Using a transgenic approach, we imaged *in vivo* in real time the behaviour of an ERV for the first time. Transgenesis entails random insertion of the transgene in the genome, which in some cases can disrupt neighbouring gene expression or induce silencing of the transgene (Akitake et al., 2011). Our initial ERV transgene Tg(*ltr5*:GFP:*ltr3*)*sh500* was engineered to resemble the ERV structure and keep both LTR sequences. However, this line started to show signs of genetic instability and silencing from the F2 generation. It is possible that the location of the insertion triggered silencing of the transgene; however, we suspect that because of the similarity to a retrovirus genome, the transgene was targeted by epigenetic mechanisms specific to ERV. Indeed, transcriptional repression of ERV has been extensively studied and key histone and DNA methylation modifications identified (Maksakova et al., 2008; Rowe et al., 2010; Turelli et al., 2014), which could be targeting the LTR promoter we used. Previous work on *zferv* identified amplification of the *zferv* locus initially in T cells from an acute lymphoblastic leukaemia zebrafish model and, although at a lesser rate, also in normal T cells (Frazer et al., 2012). We suspect that genomic amplification of the initial transgene *ltr5*:GFP:*ltr3* would be facilitated in the initial sh500 transgenic line as the transgene would be subjected to the transposase activity of other functional ERVs. This, in turn, might have activated targeting and silencing of the transgene within a generation. Either or both of those mechanisms might explain why we still observed some mild (neuron-specific) silencing phenotypes in the second transgenic line using a less-recognisable ERV structure, with the 3′LTR sequence replaced by a normal polyA tail in Tg(*ltr5*:GFP:*pA*)*sh533*.

### The role of ERVs in T lymphocytes

Despite stability issues of the transgene, we consistently observed the signature expression of *zferv* in lymphocytes, as already identified in zebrafish transcriptional studies by others (Frazer et al., 2012; Shen and Steiner, 2004). The role of ERV expression in T cells is still under investigation. Each step of T-cell differentiation is accompanied by a sequence of wide transcriptional changes that allows (1) commitment to the T-cell lineage, (2) T-cell receptor (TCR) rearrangement and (3) positive or negative selection (Mingueneau et al., 2013). It is possible therefore that transcriptional de-repression of neighbouring genes allows ERV to be expressed during those stages, which would explain such a strong expression during those early phases of T-cell development.

Whether ERV expression is merely a side effect of neighbouring gene activation or plays a crucial role during T-cell development is an important question to answer. ERVs do confer advantages to our adaptive immune system, with important implications for infectious and cancer therapeutic approaches. T cells that have been presented with ERV peptides during their initial thymic selection have a higher sensitivity to exogenous retrovirus infection, conferring an advantage in fighting HIV (Young et al., 2012). Furthermore, very recently T-cell populations were found with the ability to recognise the antigen of multiple HERVs in patients with haematological cancers, with HERV-T cell less present in healthy donors (Saini et al., 2020). This finding might lead to selective therapies using HERV as a target to kill tumour cells.

### Relevance of neuronal expression of ERV for human studies

Here, we provide evidence for a member of the ERV family being expressed in the healthy developing brain of a vertebrate. Reporter expression suggests that this neuronal expression might be more easily silenced than in T cells; the relevance of this finding for endogenous *zferv* expression, and its physiological significance, remain to be investigated. The role of endogenous retrovirus expression in developing neurons deserves further investigation as the potential role of ERVs in neurodegenerative and neurological diseases is a subject under intense study. The implications of aberrant presence of ERVs in the brain in human neurological diseases are actively being investigated, with the human HERV-K found to be upregulated in cortical neurons of amyotrophic lateral sclerosis (ALS) and MS patients (Li et al., 2015; Mameli et al., 2007; Perron et al., 1997), with currently active clinical trials targeting ERVs with anti-retroviral therapies for ALS, MS and the autoinflammatory disease AGS (Gold et al., 2018, 2019; Rice et al., 2018).

Our finding in embryos and adults suggests a role for ERVs in young neurons, potentially highlighting a role for ERVs in neuron development and maturation. There is evidence that other types of retroelements are expressed in neurons where they might function as regulators of progenitor differentiation. Indeed, brain expression of other retroelements has previously been reported, mainly focusing on the long interspersed nuclear element L1 family of non-LTR elements, which makes up 17% of all genomic DNA, constituting the largest percentage of the human genome of all retroelements (Scott et al., 1987). In the brain, L1 elements are de-repressed to allow neuronal precursor cells to differentiate and contribute to individual somatic mosaicism in human (Coufal et al., 2009) and mouse (Muotri et al., 2005). The latest study also used an engineered reporter approach by tagging GFP to the retroelement L1 (Muotri et al., 2005). Although L1 elements are not related to the ERV that is the subject of our study, it was recently discovered that additional groups of retroelements are enriched in specific regions of the human brain, in particular, LTR elements in the cerebellum (Bogu et al., 2019 preprint). With the advances in single-cell sequencing, it is possible that expression of different groups of ERVs, specifically human ERVs, will also be identified in the brain to begin a complete understanding of the presence and role of ERVs in brain development and diseases (Evans and Erwin, 2020).

### Conclusions and future work

The zebrafish has been successfully used to model human neurodegenerative and neurodevelopmental disorders. Early time points of brain development, which are particularly difficult to study in mammals, are studied with ease in the zebrafish because of its transparency. As such, zebrafish models of neurodevelopmental autoinflammatory disorders (Hamilton et al., 2020; Haud et al., 2011; Kasher et al., 2015) or neurodegenerative diseases, such as MS (Kulkarni et al., 2017) or ALS (Da Costa et al., 2014), could be combined with our *zferv1a* reporter line to elucidate the role of ERVs in the development of these diseases.

This study describes the first zebrafish reporter line for an ERV, providing a tool for investigating how certain pathological states activate ERV expression in specific cell lineages, using a real-time *in vivo* approach.

## MATERIALS AND METHODS

### Zebrafish husbandry

We used the following transgenic lines: Tg(*mpeg1*:mCherryCAAX)*sh378* to label the membrane of macrophages and microglia (Bojarczuk et al., 2016), Tg(*lck*:mCherry) to label T cells (Langenau et al., 2004) and NK cells (Hernández et al., 2018), Tg(*CD4*:mCherry) to label CD4^+^ T cells and some macrophage/dendritic cells (Dee et al., 2016) and the Tg(*lyz:nfsb-*mCherry)*sh260* for neutrophils (Buchan et al., 2019).

All zebrafish were raised in the Bateson Centre at the University of Sheffield in UK Home Office-approved aquaria and maintained following standard protocols (Nüsslein-Volhard and Dham, 2002). Tanks were maintained at 28°C with a continuous re-circulating water supply and a daily light/dark cycle of 14 h/10 h. All procedures were performed on embryos younger than 5.2 dpf, which were therefore outside of the Animals (Scientific Procedures) Act, to standards set under AWERB (Animal Welfare and Ethical Review Bodies) and UK Home Office-approved protocols.

### Identification of *zferv* genomes

We used the published full *zferv* nucleotide sequence (AF503912) as a query for a nucleotide BLAST search on zebrafish genome assembly on the Ensembl website (http://www.ensembl.org/Danio_rerio/Tools/Blast?db=core), using the most recent reference genome (GRCz11, July 2017), choosing the ‘BLAT’ option favouring highly similar sequences, and disabling the filter for species-specific repeats. We selected only hits with homology found in both the *gag*/*pol* and the *env* regions. Subsequent sequence alignments of LTRs, repetitive elements and open reading frames, and annotations were performed using DNA Strider 2.0.

### *zferv1a* full genome cloning

The full *zferv1a* sequence was subcloned from fosmid 1930h03 (Sanger Center, Cambridge, UK) predicted to contain a ∼37 kb of chromosome 4 encompassing the sequence, according the annotations of the zv9 version of the zebrafish genome (Ensembl.org). The sequence, plus ∼100 bases of flanking genomic sequence on each side, was amplified using primers 5′-CCCTGCTCATTCAACACCATAC-3′ and 5′-CCCGTCTGTGAATTACCAAGC-3′, and cloned into PCR4-TOPO (Thermo Fisher).

### RNA *in situ* hybridisation

The *env1a* probe was made from the plasmid containing the full *zferv1a* genome with the following primers: BamH1_env1a_fwd, TGGGGATCCATGAATAAAATAAACAAATTGG, and SmaI_env1a_rev, CCACCCGGGCACCATATCCAATAGTTCCTCC, generating a 1935 bp (*env1a*) fragment. The *pol1a* probe was made from the same plasmid using BamH1_pol1a_fwd, TGGGGATCCGGCAGCAGACGCCGCTGCTA, and Xba1_pol1a_rev, TGGTCTAGACCTCAGGCTCCTCAGTGTCT, generating a 1295 bp fragment. The *env1b* probe was made from cDNA using BamH1_env1b_fwd, TGGGGATCCatgaATATAAACAAATTGGTGG, and SmaI_env1b_rev, CCACCCGGGCAGAACGCTATAGTCAGTGCTC, generating a 635 bp (*env1a*) fragment. Fragments were subsequently cloned into Zero Blunt™ TOPO™ vector (Invitrogen) and reverse transcribed into RNA using the DIG labelling kit (Roche) (SP6 enzyme for antisense probe and T7 for sense probe). Whole-mount *in situ* hybridisation on wild-type *nacre* zebrafish was performed to identify the spatial and temporal pattern of *zferv1a* expression using the envelope (*env*) probe *env1a* and the reverse transcriptase probe *pol1a* and from *zferv1b* using the envelope probe *env1b*. We fixed wild-type embryos from the *nacre* strain at different stages and followed an *in situ* hybridisation protocol as previously described (Thisse and Thisse, 2008).

### Generation of *zferv1a* and *lck:mCherry* transgenic lines

LTR5 and LTR3 regions of *zferv1a* were cloned into p5E and p3E Gateway entry vectors, respectively, using the following primers: BamH1_LTR3′_fwd, TGTGGATCCTCTCTTTCGAGATCAAGAGAGGG; Xho1_LTR3′_rev, ACACTCGAGGCTGCACCTTGTTGGAAAATGG; kpn1_LTR5′_fwd, TGTGGTACCCCTCTCTCTTGTAAAGGTTGAGGG; and sacII_LTR5′_rev ACACCGCGGTTTAATAATGGTTTTGTCTCCC.

An LR reaction was performed into the pDestCRY-CFP vector, which was injected into one-cell-stage zebrafish embryos to create the following transgenic lines: Tg(*ltr5:*GFP*:ltr3*)*sh500* and Tg(*ltr5:*GFP*:pA*)*sh533*, subsequently called Tg(*zferv1a:*GFP)*sh533.*

The previously described *lck* promoter (Langenau et al., 2004) was used to create the transgene *lck*:mCherry in the pDestCRYmCherry vector by Gateway recombination. Stable lines were created by injecting these constructs with Tol2 mRNA in one-cell-stage embryos and selecting founders using the red eye marker.

### Tyrosinase CRISPR/Cas9 crispant generation

Synthetic SygRNA (crRNA and tracrRNA) (Merck) in combination with Cas9 nuclease protein (Merck) was used for gene editing. Transactivating RNAs (tracrRNA) and gene-specific CRISPR RNAs (crRNA) were resuspended to a concentration of 20 µM in nuclease-free water containing 10 mM Tris-HCl pH 8. SygRNA complexes were assembled on ice immediately before use using a 1:1:1 ratio of crRNA:tracrRNA:Cas9 protein. Gene-specific crRNAs were designed using the online tool CHOPCHOP (http://chopchop.cbu.uib.no/). Tyrosinase crRNA tyr: GGACTGGAGGACTTCTGGGG(AGG) (Isles et al., 2021).

### Flow cytometry analysis

The head kidney of three adult Tg(*zferv1a:*GFP)*sh533* and three wild-type *nacre* fish were dissected and collected into cold Leibovitz 15 media supplemented with 20% foetal bovine serum and 0.5 mM EDTA. The tissue was pipetted up and down to create a single-cell suspension and kept on ice. Negative gate was set up using cells from *nacre* animals with the Blue530 detector on an FACSARIA IIu flow cytometry machine and all analysis carried out using the FlowJo software. Gating was performed as described in Fig. S5 using the GFP-positive population back-gated onto the different cell type populations: monocytes, progenitors, T cells and erythrocytes.

### Colocalisation imaging and analysis

Tg(*zferv1a:*GFP)*sh533* was crossed to Tg(*lck*:mCherry), Tg(*CD4*:mCherry), Tg(*lyz:nfsb-*mCherry)*sh260* and Tg(*mpeg1*:mCherry:CAAX)*sh378*, and double-positive animals were selected and imaged at 5 dpf using the Perkin Elmer Spinning Disk system for 3 h. Whole *z*-stacks were imported into Fiji to perform colocalisation analysis using the Colocalisation Threshold Plugin. Whole *z*-stacks at three different time points during the time lapse in four different larvae were used with the same threshold values to determine the colocalisation coefficient by Pearson's correlation.

### Single-cell RNA-sequencing dataset analysis

Expression of *zferv1a* (*ENSDARG00000110878*) in different haematopoietic lineages was obtained from single-cell RNA-sequencing databases of seven different transgenic lines each labelling a different lineage (Athanasiadis et al., 2017). Single-cell RNA-sequencing raw counts files of zebrafish blood cells were obtained from datasets available on Array Express (https://www.ebi.ac.uk/arrayexpress/; accession codes: E-MTAB-5530 and E-MTAB-4617). The data were then processed and analysed using the Seurat software package for R (Hao et al., 2021; Satija et al., 2015). The data were selected to have a minimum of 200 genes in each cell and then merged and normalised. The Seurat wrapper fastMNN was used to correct for batch effects and uniform manifold approximation and projection (UMAP) analysis was performed on the resulting combined dataset, which resulted in nine clusters. The function ‘FindAllMarkers’ was used to find the differentially expressed genes in each cluster and these were compared with marker genes published in the studies to assign cluster identity. Expression distributions across clusters were obtained for ENSDARG00000110878. Differential expression of CD4^+^ cells and T cells versus all cells was determined (average log2 fold change=1.98, adjusted *P*-value=5.31e−81).

Statistical differential expression was performed in Seurat between each cluster with Mann–Whitney test followed by a Kruskal–Wallis multiple testing correction.

### *zferv1a* overexpression

The plasmid containing the full *zferv1a* gene was injected in equimolar concentration alongside a plasmid containing GFP into one-cell-stage zebrafish embryos, using embryos injected with GFP only as control. RNA was extracted from 30 hours post-fertilisation GFP-positive embryos from both groups using Trizol and cDNA was synthesised as previously described (Hamilton et al., 2020). qPCR was performed to measure the level of expression of GFP and *zferv1a* normalised to the *ef1a* reference gene using the following primers: egfpforward, CCATCTTCTTCAAGGACGAC; egfpreverse, CGTTGTGGCTGTTGTAGTTG; ZFERVpol-1S, GCTAGGACATCCCATTGTGT; ZFERVpol-2A, GGGAATGTGTTCTGGTGTCT; Ef1a-1S, GCTGATCGTTGGAGTCAACA; Ef1a-2A, ACAGACTTGACCTCAGTGGT.

### Statistical analyses

All statistical analyses were performed in GraphPad Prism where data was entered using a grouped table for ANOVA analysis (more than two samples or two variables). Sample distribution was assessed using frequency of distribution analysis. *n* (experimental unit) number for each experiment is stated in figure legends. *P*-values are indicated in legends and asterisks used for graphs with multiple comparisons: *P<0.05, **P<0.01, ***P<0.001, ****P<0.0001. Following the recommendation of the American Statistical Association, we do not associate a specific *P*-value with significance (Wasserstein et al., 2019).

## Supplementary Material

Supplementary information
