# Supplementary material for: A zebrafish reporter line reveals immune and neuronal expression of endogenous retrovirus
Source: Dis Model Mech. 2022 Apr 13;15(4):dmm048921. doi: 10.1242/dmm.048921 (PMC9016899; doi:10.1242/dmm.048921)
Supplement: Supplementary information [file dmm-15-048921-s1.pdf]

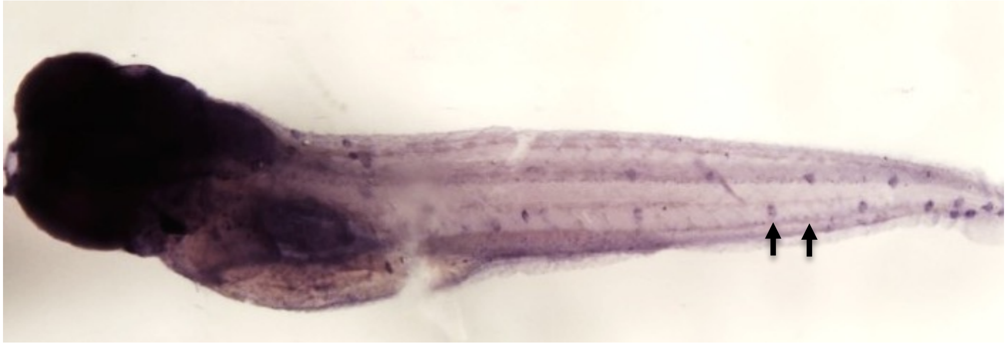

Fig. S1. Long development of the *env* probe from *zferv1a* by ISH revealed labelling of neuromasts (Black arrows).

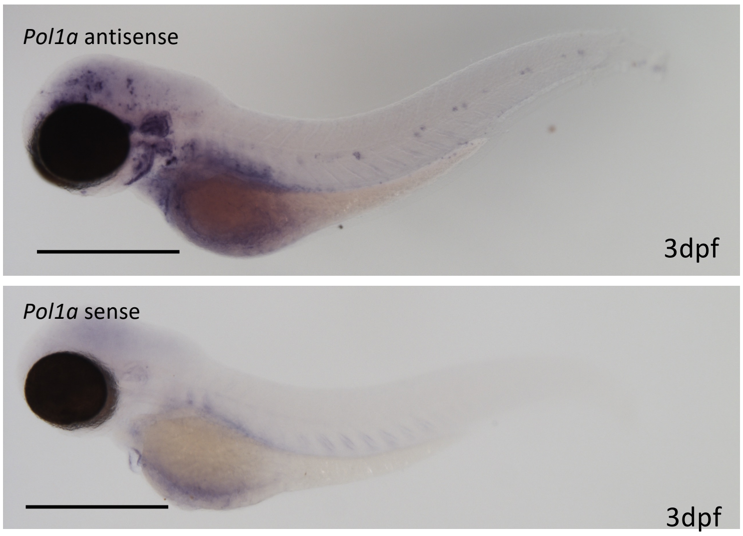

Fig. S2. Expression of the reverse transcriptase gene (*pol*) of *zferv1a* (called *pol1a* here) by *in situ* hybridisation at 3dpf. Scale bar 500μm.

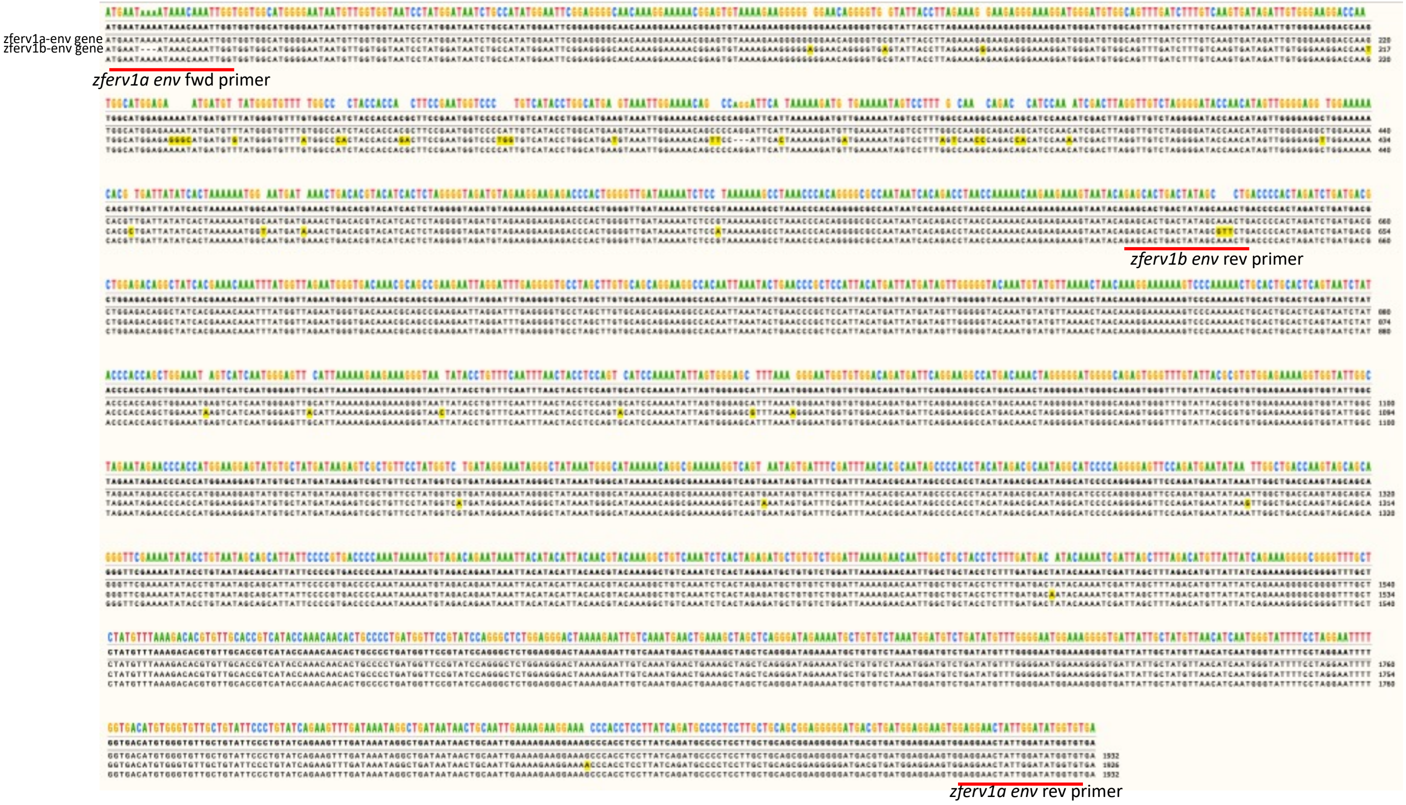

Fig. S3. Alignment of the envelope gene from zferv1a and zferv1b to highlight to regions with significant differences between the two genes. Primers used to design the probes are underlined in red.

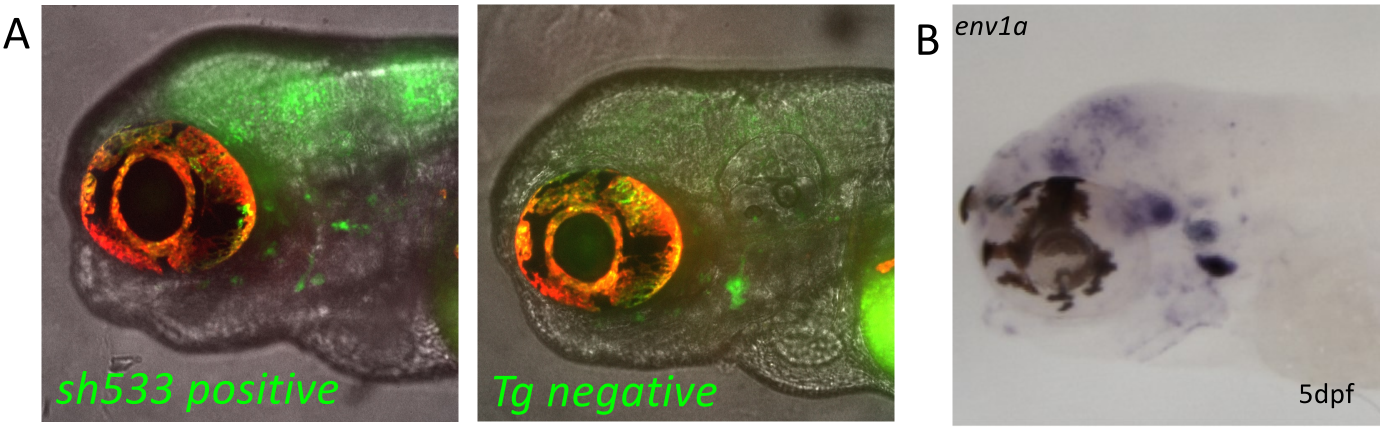

Fig. S4. A. *sh533* positive and negative embryo illuminated with the GFP and mCherry laser to highlight the similar light reflection in the lenses that act as mirrors. **B. ISH with RNA probe env1a in a 5dpf embryos injected with a crRNA against the tyrosinase gene generating a mosaic pattern of pigment in the eye**

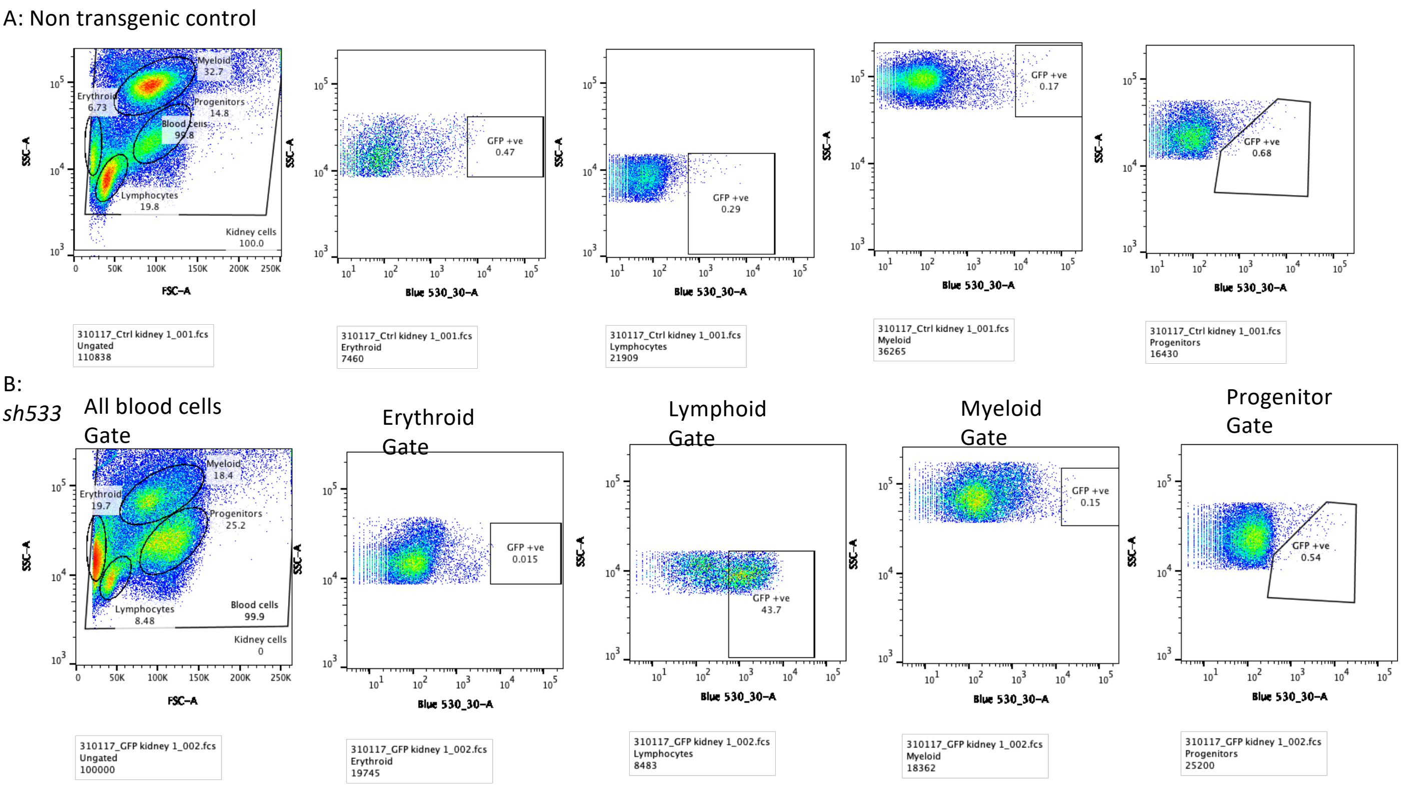

Fig. S5. Gating strategy for each heamatopoietic lineages (erythroid, lymphoid, myeloid and progenitor) drawn first in non transgenic control (A) and applied to the sh533 transgenic kidney samples.

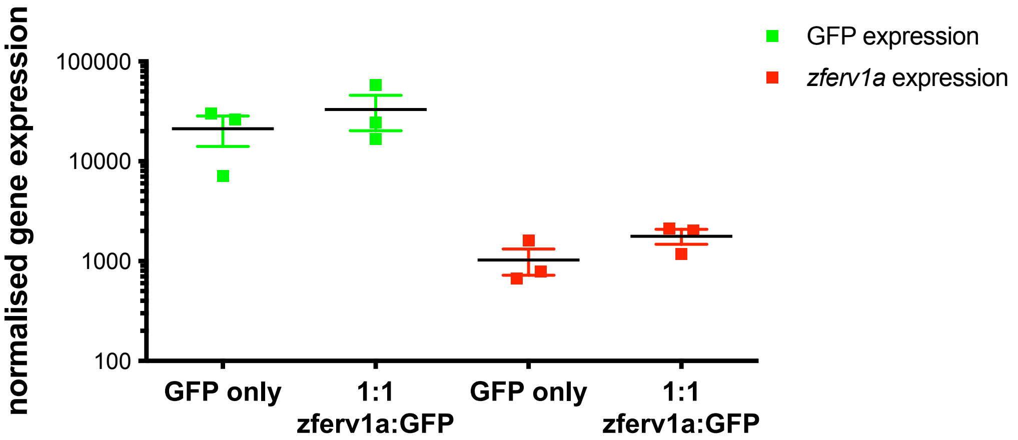

Fig. S6. Expression of GFP (green) and zferv1a (red) by qPCR after 1-cell stage injection of a GFP expressing vector alone (GFP only) or in equimolar concentration with zferv1a expression vector (1:1 zferv1a:GFP).

Disease Models & Mechanisms • Supplementary information

Table S1. Coordinates of the *zferv1-7* genomic segments

ERV name	chromosome (scaffold)	orientation	5' limit	3' limit
zferv1a	4	reverse	62,546,461	62,557,677
zferv1b	KN149691.1 - NW_001884427.4	forward	211.988	223.087
zferv2	7	reverse	19,379,508	19,389,176
zferv3	16	reverse	9,986,975	9,996,169
zferv4	5 - NW_018394575	reverse	168.525	178.11
zferv5	15	forward	44,144,056	44,162,279
zferv6	7 - NW_018394672	reverse	116.062	124.356
zferv7	19	forward	7,960,461	7,968,734

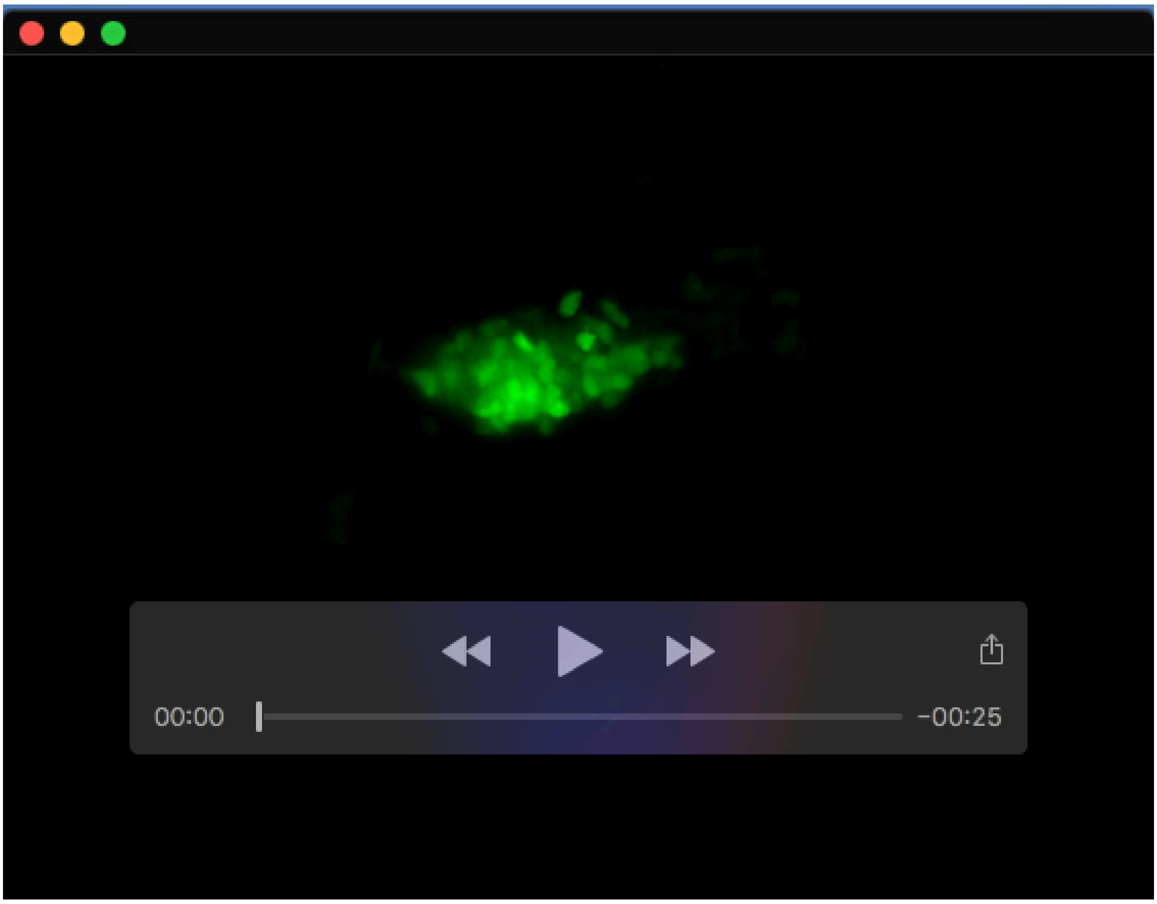

Movie 1. Timelapse using lightsheet microscopy of sh533 transgenic embryos at 5dpf, duration 1h30 every 50sec as fast as possible. 215um stack every 1um slice.

Disease Models & Mechanisms • Supplementary information
